# Evaluation of attitudes and knowledge toward mental disorders in a sample of the Chinese population using a web-based approach

**DOI:** 10.1186/s12888-018-1949-7

**Published:** 2018-11-20

**Authors:** Juan Li, Meng-meng Zhang, Lin Zhao, Wen-qiang Li, Jun-lin Mu, Zhao-hui Zhang

**Affiliations:** 1 The Second Affiliated Hospital of Xinxiang Medical University, 388# Jianshe Road, Muye, Xinxiang, China; 2 Xinxiang Medical University, 601# Jinsui Road, Hongqi, Xinxiang, China; 3 Henan Key Laboratory of Biological Psychiatry (Xinxiang Medical University), 388# Jianshe Road, Muye, Xinxiang, China; 4 Xinxiang Key Laboratory of Electrophysiology, 388# Jianshe Road, Muye, Xinxiang, China

**Keywords:** Mental health knowledge, Stigma, Discrimination, Mental disorders, China

## Abstract

**Background:**

People with mental disorders often encounter stigmatizing attitudes related to their conditions. Stigma often represents one of the critical obstacles that stand in the way of delivering mental health care. The main aim of the study was to assess the knowledge and attitudes toward mental disorders in a sample of the Chinese population; furthermore, we also aimed to identify and explore the socio-demographic characteristics associated with specific knowledge and attitudes towards psychiatric disorders.

**Methods:**

A cross-sectional survey was created and delivered through an Internet chat application over the period June–December 2017. The Mental Health Knowledge Questionnaire and the Perceived Devaluation and Discrimination Scale were used to evaluate the participants’ mental health knowledge and attitudes toward mental disorders.

**Results:**

A total of 1087 participants were recruited in for our survey. The mean score of the MHKQ and PDD were (15.89 ± 2.69) and (33.77 ± 6.66), respectively. Univariate analyses showed that young people and rural residents tended to show more positive attitudes toward mental disorders with respect to older people and urban residents (*P* < 0.05). People with higher education levels, those who had contact with people with mental disorders, and those who learned about mental disorders by personal encounter resulted to have had higher MHKQ scores (*P* < 0.05).

**Conclusions:**

In our sample of the Chinese population, negative attitudes toward mental disorders were often reported. General education programs may not be an effective way to decrease stigma, while anti-stigma campaigns targeted for specific groups, such as urban residents and the older people, should be carried out in the future in China.

## Background

Although there are now a variety of effective treatments for mental disorders, still a significant number of people do not receive proper treatment for mental disorders [1]. Even those who receive care and assistance from mental health services often tend to exhibit a significant delay between symptoms’ onset and seeking treatment and this can exacerbate their conditions and increase the psychological and financial burdens [2]. The fear of being discriminated against has been reported to play a significant role in creating barriers and increasing the delay in seeking treatment [3]. Stigma and discrimination are widely experienced by people with mental disorders in many domains of their daily life, such as in employment, social activities, personal relationships, housing, marriage, and so on [4–8]. In China, words like “violent,” “crazy,” “strange”, and “useless” are often used to describe people with mental disorders [9, 10]. Stigma and discrimination surrounding mental disorders represent not only factors that hamper treatment-seeking [11], but may also delay the healing process for people with mental disorders [12] and prevent people with mental disorders from achieving their social rights and full participation in the life of their community.

Moreover, the family members of people with mental disorders are often blamed by the public [13]. Empirical evidence showed that families of people with mental disorders are likely to ignore the problem when their family members experience discrimination: they may hide them from public life, delay treatment seeking, or even reject professional help. Such attitudes and behaviors may determine a pejorative illness course and increase the psychological and financial burdens on the families [14–16].

In order to reduce the treatment gap and provide more consistent and accessible mental health services, the World Health Organization (WHO) has encouraged countries to integrate mental health services into their primary care systems. However, negative attitudes toward mental disorders among the general population are likely to represent an obstacle to the development of more efficient community mental health services [17].

Additionally, the general public could, through greater social engagement and more acceptance of mental disorders, play an essential role in rehabilitating the mentally ill. This is no easy task, since people with mental disorders have often been labeled as “violent” and “dangerous”, and the attitudes of the general public have contributed to exacerbating the conditions of people with mental disorders [18].

Thornicroft et al. [19] defined stigma as an overarching term that refers to three main elements: problems of knowledge (ignorance), problems of attitude (prejudice), and problems of behavior (discrimination). It has been reported that lack of accurate mental health knowledge may be one of the leading factors that may contribute to stereotyping people with mental disorders [20]. However, it has also been found that health professionals, who are supposed to have greater knowledge concerning mental health issues with respect to the general population, often have more negative attitudes toward mental disorders than the general public [21–23]. This finding contradicts a common assumption that greater knowledge of mental disorders results in less of a discriminatory attitude, and thus it throws into question the, supposedly positive, relationship between mental health knowledge and discriminatory attitudes.

The main aims of this study were to evaluate attitudes and knowledge about mental disorders in a sample of the Chinese general population and to explore their relationships with socio-demographic characteristics. Results from this study could yield some applications: first, it could help in designing programs that would aim to reduce public stigma against mental disorders; second, it could provide guidance for the government to undertake further strategic action.

## Methods

### Participants

A “snowball” sampling method was used to recruit participants. A digital version of a self-made schedule and of two standardized questionnaires was sent to 50 people known to the study authors (including families, friends, and other acquaintances), who previously agreed to participate in the study and to share the digital questionnaires through the Internet chat application “We-Chat”, which has over 800 million active members in China. A standardized statement accompanying the questionnaire encouraged all the participants to transmit the electronic questionnaire to their friends or family members. Participants were informed, at the beginning of the survey, that expression “mental disorders” in the questionnaire referred to schizophrenia, depression, and bipolar disorder. All potential participants were also informed that they had a chance to win a raffle prize as a reward.

Participants were finally asked to provide anonymous informed consent in electronic format, before taking part in the survey. The survey was conducted from June to December 2017. The study protocol was approved by the Research Ethics Committee of the Second Affiliated Hospital of Xinxiang Medical University.

### Instruments

A self-made questionnaire was used to collect participants’ basic demographic data (including age, gender, level of education, and place of residence).

The Mental Health Knowledge Questionnaire (MHKQ) was developed to evaluate public knowledge and awareness of mental health by the Chinese Ministry of Health (MOH) in 2009. It contains 20 self-administered items. Items 1–16 (the first section) require participants to select “true,” “false,” or “unknown” about statements concerning mental health. For items 1, 3, 5, 7, 8, 11, 12, 15 and 16, a “true” answer corresponded to a 1-point score, while a “false” or “unknown” answer corresponded to score of 0. By contrast, for items 2, 4, 6, 9, 10, 13 and 14, a “false” answer gave a score of 1, while “true” or “unknown” answers corresponded to score of 0. Finally, items 17–20 (the second section) are statements concerning previous knowledge about the “four mental health promotion days”. Total scores range from 0 to 20, with higher scores indicating greater knowledge of mental health issues. The Cronbach’s coefficient of MHKQ was reported to be 0.61 [24].

The Perceived Devaluation and Discrimination Scale (PDD) [25] was used to assess the degree of stigmatizing attitudes toward people with mental disorders. It contains 12 items and each item is rated on a 5-point scale, ranging from 1 (totally agree) to 5 (totally disagree). Items 1, 2, 3, 4, 8 and 10 required reverse scoring. Total scores ranged from 12 to 60, with higher scores indicating lower levels of stigma. The Chinese version of the PDD has been reported to have strong internal consistency (Cronbach’s α = 0.70).

Participants were also requested to answer three additional questions concerning their source of information about mental disorders (i.e., portrayals in mass media vs. direct encounter), level of contact with mentally ill people, and their attitude toward psychotropic drugs (with the following four response options: “Effective”, “They make people worse”, “Ineffective” and “Likely to lead to dependency”).

### Data analysis

Descriptive statistics were used to explore basic socio-demographic data. The scores obtained from PDD and MHKQ were then compared among sample subgroups, created according to demographic characteristics, using one-way analysis of variance. The demographic information used to create subgroups included gender, age, education, place of residence, sources of information, and level of contact with people with mental disorders. The correlation between knowledge about mental disorders and attitudes toward those disorders was examined by the Pearson’s correlation coefficient. For all statistical analyses SPSS v18 was used and the level of significance was set at *P* < 0.05.

## Results

### Sample characteristics

A total of 1104 participants finished the questionnaires, but 15 participants were excluded because they reported having been diagnosed with mental disorders in the past and two participants were excluded because they were below legal age for providing informed consent (i.e., 16 years). A total of 1087 participants were finally included in our survey and their mean (±SD) age was 33.93 (±9.76) years, within a range of 16–67 years. Our participants were predominantly female (*N* = 693; 63.8%). The socio-demographic characteristics are summarized in Table 1.

**Table 1:** Characteristics of the participants (*n* = 1087)

Variable	N	%
Gender		
Male	394	36.2
Female	693	63.8
Age (years)		
16–24	114	10.5
25–34	558	51.3
35–44	235	21.6
45 and above	180	16.6
Education		
Junior high school or less	97	8.9
Senior high school	160	14.7
College degree/undergraduate	608	55.9
Postgraduate or above	222	20.4
Place of residence		
Urban area	901	82.9
Rural area	186	17.1

### Contact with and general knowledge about mental disorders

The majority of our respondents declared previous contact with people suffering from mental disorders (64.9%; *n* = 706), although 59.7% (*n* = 649) of participants reported that they learned about mental disorders from portrayals in mass media, while 40.3% (*n* = 438) had personal encounters with mentally ill people as a primary source of knowledge about mental disorders. With respect to the participants’ attitudes toward psychotropic drugs, 66.9% (*n* = 727) of them considered them effective, 3.7% (*n* = 40) of participants believed that they would lead to a worse outcome, 25.8% (*n* = 280) envisaged the risk to lead to dependency, and 3.7% (*n* = 40) considered them “ineffective”.

### Responses frequencies for the PDD and MHKQ

The mean total score for the PDD was 33.77 (SD = 6.66), and scores ranged from 12 to 57. The answer for each item is displayed in Table 2.

**Table 2:** The results of PDD (*n* = 1087)

Items	Strongly agree (n, %)	Agree (n, %)	Unsure (n, %)	Disagree (n, %)	Strongly disagree (n, %)
1. Most people would accept a former mental patient as a close friend.	49 (4.5)	149 (13.7)	545 (50.1)	253 (23.3)	91 (8.4)
2. Most people believe that a person who has been in a mental hospital is just as intelligent as the average person.	123 (11.3)	300 (27.6)	418 (38.5)	187 (17.2)	59 (5.4)
3. Most people believe that a former mental patient is just as trustworthy as the average citizen.	92 (8.5)	259 (23.8)	422 (38.8)	249 (22.9)	65 (6.0)
4. Most people would accept a fully recovered former mental patient as a teacher of young children in a public school.	98 (9.0)	285 (26.2)	286 (26.3)	258 (23.7)	160 (14.7)
5. Most people believe that entering a mental hospital is a sign of personal failure.	23 (2.1)	120 (11.0)	213 (19.6)	357 (32.8)	374 (34.4)
6. Most people would not hire a former mental patient to take care of their children, even if he or she had been well for some time.	360 (33.1)	421 (38.7)	169 (15.5)	96 (8.8)	41 (3.8)
7. Most people think less of a person who has been in a mental hospital.	116 (10.7)	397 (36.5)	256 (23.6)	233 (21.4)	85 (7.8)
8. Most employers will hire a former mental patient if he or she is qualified for the job.	73 (6.7)	198 (18.2)	455 (41.9)	268 (24.7)	93 (8.6)
9. Most employers will pass over the application of a former mental patient in favor of another applicant.	199 (18.3)	496 (45.5)	256 (23.6)	101 (9.3)	35 (3.2)
10. Most people in my community would treat a former mental patient just as they treat anyone.	83 (7.6)	311 (28.6)	440 (40.5)	209 (19.2)	44 (4.0)
11. Most young women would be reluctant to date a man who has been hospitalized for serious mental disorder.	365 (33.6)	443 (40.8)	208 (19.1)	53 (4.9)	18 (1.7)
12. Once they know a person was in a mental hospital, most people will take his or her opinions less seriously.	124 (11.4)	430 (39.6)	342 (31.5)	162 (14.9)	29 (2.7)

As for the MHKQ, the total scores ranged from 5 to 20 with a mean score of 15.89 (±2.69). The mean score for the first section (true vs. false questions) was 13.50 (± = 2.17), and 159 (14.6%) participants got a score of 16.

For the second section of MHKQ, there were 258 (23.7%) participants who recognized all four mental health promotion days. The rate of correct responses is displayed in Table 3.

**Table 3:** The correct response rate of MHKQ (*n* = 1087)

Item	n	Percent (%)
1. Mental health is a component of health. (true)	1047	96.3
2. Mental disorders are caused by incorrect thinking. (false)	596	54.8
3. Many people have mental problems but do not realise it. (true)	1052	96.8
4. All mental disorders are caused by external stressors. (false)	653	60.1
5. Components of mental health include normal intelligence, stable mood, a positive attitude, quality interpersonal relationship and adaptability. (true)	1035	95.2
6. Most mental disorders cannot be cured. (false)	742	68.3
7. Psychological or psychiatric services should be sought if one suspects the presence of psychological problems or a mental disorder. (true)	969	89.1
8. Psychological problems can occur at almost any age. (true)	1049	96.5
9. Mental disorders and psychological problems cannot be prevented. (false)	825	75.9
10. Even for severe mental disorders (eg, schizophrenia), medications should be taken for a given period of time only; there is no need to take them for a long time. (false)	887	81.6
11. Positive attitudes, good interpersonal relationships and healthy life style can help maintain mental health. (true)	1046	96.2
12. Individuals with a family history of mental disorders are at a higher risk for psychological problems and mental disorders. (true)	981	90.2
13. Psychological problems in adolescents do not influence academic grades. (false)	964	88.7
14. Middle-aged or elderly individuals are unlikely to develop psychological problems and mental disorders. (false)	957	88.0
15. Individuals with a bad temperament are more likely to have mental problems. (true)	838	77.1
16. Mental problems or disorders may occur when an individual is under psychological stress facing major life events (eg, death of family members). (true)	1031	94.8
17. Have you heard about International Mental Health Day? (yes)	613	56.4
18. Have you heard about the International Day against Drug Abuse and Illicit Drug Trafficking? (yes)	947	87.1
19. Have you heard about the International Suicide Prevention Day? (yes)	345	31.7
20. Have you heard about World Sleep Day? (yes)	694	63.8

### PDD and demographic variables

Table 4 shows the mean PDD scores for different subgroups of participants, stratified according to basic socio-demographic characteristics. There were statistically significant differences in the scores that matched with age and place of residence. Analysis of variance showed that those under 25 years old scored higher than those above that age, but the difference was significant only with respect to those aged 35–44 years (*F* = 5.37, *P* = 0.001). Participants from rural areas scored significantly higher than those from cities (*t* = − 2.42, *P* = 0.016). Differences in the stigma level according to participants’ gender, educational level, sources of information about mental disorders, and contact levels with people with mental disorders were not statistically significant.

**Table 4:** Associations between PDD and demographic variables

Item	n	Mean score	t/F	*P*
Gender			0.67	0.500
Male	394	33.96 ± 7.24		
Female	693	33.66 ± 6.31		
Age (years)			5.37	0.001
16–24	114	35.11 ± 5.48		
25–34	558	34.03 ± 6.69		
35–44	235	32.37 ± 7.11		
45 and above	180	33.92 ± 6.39		
Education			0.13	0.942
Junior high school or less	97	33.90 ± 6.76		
Senior high school	160	33.82 ± 6.77		
College/undergraduate	608	33.83 ± 6.58		
Postgraduate or above	222	33.52 ± 6.79		
Place of residence			−2.42	0.016
Urban area	901	33.56 ± 6.74		
Rural area	186	34.78 ± 6.20		
Sources of information about mental disorders			1.31	0.192
Mass media	649	33.99 ± 6.44		
Personal encounters	438	33.44 ± 6.97		
Contact level			1.40	0.161
No	381	34.15 ± 6.54		
Yes	706	33.56 ± 6.72		

### MHKQ and demographic variables

Table 5 shows the mean MHKQ scores for different demographic groups of participants. The impact of participants’ ages, educational level, areas of residence, sources of information about mental disorders, and level of contact with mentally ill people was significant over the MHKQ scoring. The analysis of variance showed that those aged 25–44 years had significantly higher MHKQ total scores than those above 45 years old. Participants with higher education levels had higher MHKQ scores (*F* = 65.72, *P* < 0.001). Urban residents had higher MHKQ scores than rural residents (*t* = 7.32, *P* < 0.001). Those who had previous contact with people with mental disorders (*t* = − 4.85, *P* < 0.001) and who had learned about mental disorders with personal encounters (*t* = − 2.10, *P* = 0.036) had higher MHKQ scores.

**Table 5:** Associations between MHKQ and demographic variables

Item	n	Mean score	t/F	*P*
Gender			−1.02	0.310
Male	394	15.78 ± 2.80		
Female	693	15.95 ± 2.63		
Age (years)			4.56	0.003
16–24	114	15.82 ± 2.16		
25–34	558	15.93 ± 2.86		
35–44	235	16.27 ± 2.52		
45 and above	180	15.30 ± 2.61		
Education			65.72	< 0.001
Junior high school or less	97	13.36 ± 2.28		
Senior high school	160	14.69 ± 2.36		
College/undergraduate	608	16.17 ± 2.51		
Postgraduate or above	222	17.08 ± 2.58		
Place of residence			7.32	< 0.001
Urban area	901	16.15 ± 2.62		
Rural area	186	14.60 ± 2.65		
Sources of information about mental disorders			−2.10	0.036
Mass media	649	15.74 ± 2.54		
Personal encounters	438	16.10 ± 2.89		
Contact level			−4.85	< 0.001
No	381	15.35 ± 2.56		
Yes	706	16.18 ± 2.72		

### The relationship between PDD and MHKQ

We didn’t find any statistically significant relationship between the total scores of the PDD and MHKQ scales (*r* = − 0.032, *P* = 0.293).

## Discussion

To the best of our knowledge, this is the first study to evaluate the general public knowledge of and attitudes toward mental disorders through a web-based survey in China. Although our samples couldn’t represent the entire Chinese population, we can still provide a reliable account of attitudes and knowledge toward mental health in China. Compared with previous studies [26–28], our results showed that mental health knowledge has improved recently, while most people’s attitudes toward mental disorders are still negative. Hence, it is vital to further discuss how to improve mental health knowledge and what might be the impact of such programs on people’s attitudes toward mental disorders.

In terms of knowledge on mental disorders and stigmatizing attitudes, we didn’t find any differences with respect to participants’ gender, in line with previous studies [20, 24]. However, there were also studies that investigated such measures in medical students and mental health staff that demonstrated that women tend to have greater knowledge about mental health and that they were more willing to interact with people with mental disorders [29, 30]. In our survey, we did not report such significant differences and thus this issue should be further explored in order to properly discuss the need of developing gender-specific anti-stigma interventions in China.

In contrast to other studies [26], our study also found that residents in rural areas had more positive attitudes toward people with mental disorders than those who lived in urban areas. One possible explanation for this evidence is that rural communities may be more tolerant of unusual behaviors, typical of people with mental disorders. However, evidence from previous studies provided rather mixed results. Girma et al. [20] found that living in rural places was the strongest predictor of families holding stigmatizing attitudes toward their mentally ill relatives. Authors reported that rural residents showed significantly higher stigmatizing attitudes than urban residents, perhaps because of lower mental health literacy. However, we didn’t find a significant relationship between mental health literacy and discriminatory attitudes.

Our analyses did not yield any significant relationship between educational level and discrimination, but we found that participants with higher levels of education were more likely to have greater mental health knowledge. Some studies reported that lower education levels were more strongly associated with negative attitudes toward mental disorders [31, 32], but others found the inverse to be also true [33, 34]. These studies indicated that highly educated people had higher expectations of social responsibility and functioning than those with less education, and that they associated people with mental disorders with lower levels of responsibility and functioning.

Today, more and more educational initiatives and informational campaigns have been implemented to improve the public’s mental health knowledge, in order to change negative opinions toward people with mental disorders. Some of the interventions yielded positive results, as expected [35, 36]. But in our study, we didn’t find any relationship between mental health knowledge and stigmatizing attitudes. In accordance with other studies, our data tend to support the idea that the public’s mental health knowledge might not represent a highly effective remedy against discrimination [37]. As some studies have shown, health care staff with supposedly greater knowledge about mental health issues are likely to hold more negative attitudes toward mental disorders than the general population [21, 22]. Moreover, many people with mental disorders have been unfairly treated by health professionals when they have sought help for medical diseases [23]. Altogether, these findings suggest that other ways to decrease stigma against mental disorders are still to be pursued.

Exposing the general public to people with mental disorders has been a method used by many anti-stigma campaigns as an effective way to improve attitudes towards mental disorders among the target group [36, 38–40]. However, in our survey we didn’t find any significant difference in the attitudes of subgroups who had or had no previous contact with people with mental disorders. Participants who reported having had contact with people with mental disorders also showed greater mental health knowledge. Once again, such findings are in line with the idea that there is no correlation between knowledge of mental disorders and positive attitudes towards them.

In our study, while 64.9% of participants reported having had direct contact with people with mental disorders, most of our respondents (59.7%) acquired their understanding of mental disorders from portrayals in mass media. For people who had no direct contact with people suffering from mental disorders, mass media may be a relevant source of information which may strongly influence their perceptions and attitudes about mental disorders. However, mass media often portray people with mental disorders as dangerous, strange, unpredictable, and violent [9], which could further lead the general public to fear people with mental disorders, cause people with mental disorders to feel isolated and rejected, create greater discrimination and prevent people with mental disorders from fully integrating into society [41]. Interventions aimed at changing portrayals of mental disorders in mass media might represent a valid means to induce a significant positive change in public attitudes toward people with mental disorders.

Finally, we didn’t find an increased risk for stigmatizing attitudes except for the age and place of residence factors. A German study found that gender, age, education level, and place of residence only accounted for 1.4% of the variance [42].

There are some limitations to our study. First, the survey was based on a cross-sectional sampling strategy and on self-reported measures, so it might be difficult to make reliable inferences about the correlations or causal relationships between attitudes and knowledge. Second, since our study was a web-based survey, we couldn’t provide the participants with in-person explanations of the questions and this might influence their responses. On the other hand, using a web-based strategy allowed participants to participate in the study without feeling socially pressured or guided to respond in one way or another. Finally, since included participants represented a convenience sample, it could not fully represent the Chinese population. The latter consideration coupled with some imbalance in terms of socio-demographic characteristics of our sample (e.g., greater ratio of female respondents) might imply that our study should be replicated in a larger and more representative sample of the Chinese population.

## Conclusions

Our findings suggested that overall mental health knowledge may have improved in the Chinese population over the years, but that most Chinese people still hold negative attitudes toward mental disorders. According to our data, this tendency seems to be more relevant in urban areas. It is important to carry out anti-stigma campaigns for the future progress of mental health in China. Although some campaigns aim to improve the public’s mental health knowledge, it is still a matter of open debate whether increasing mental health knowledge may actually decrease people’s discriminatory attitudes toward people with mental disorders or not. Finally, although we did not find a positive correlation between participants’ levels of contact with people with mental illness and their attitudes toward people with mental disorders, we did find similar evidence in other studies. Thus, we should further explore the hypothesis that more frequent contact with people with mental disorders may change negative attitudes towards mental disorders.

## Abbreviations

MHKQ: The Mental Health Knowledge Questionnaire (MHKQ)
MOH: Ministry of Health
PDD: Perceived Devaluation and Discrimination Scale
WHO: World Health Organization
